# Socioeconomic and health system determinants of maternal health inequities across Chinese provinces (2009–2021): a multivariate meta-regression analysis of regional heterogeneity in underlying mechanisms

**DOI:** 10.3389/fpubh.2026.1722508

**Published:** 2026-02-17

**Authors:** Min Luo, Hua Ding, Xueting Chen, Ping Luo, Enhong Dong, Shaojie Li

**Affiliations:** 1 Shanghai East Hospital, Shanghai, China; 2 Department of Clinical Psychology, Shanghai Fourth People’s Hospital, School of Medicine, Tongji University, Shanghai, China; 3 School of Nursing and Health Management, Shanghai University of Medicine & Health Science, Shanghai, China; 4 Department of Statistics, Shanghai University of Finance and Economics Zhejiang College, Jinhua, China; 5 Digital Intelligence Management Research Institute, Shanghai University of Finance and Economics Zhejiang College, Jinhua, China; 6 Institute of Healthy Yangtze River Delta, Shanghai Jiao Tong University, Shanghai, China; 7 Outpatient and Emergency Management Office, Shanghai Sixth People's Hospital, Shanghai, China

**Keywords:** China, health system, inequity, maternal mortality rate, meta-regression, socioeconomic determinants

## Abstract

**Background:**

Despite China’s significant reduction in maternal mortality rate (MMR), substantial inter-provincial disparities persist, reflecting uneven socioeconomic development and health system capacity. Previous studies have rarely examined the interplay of these factors using standardized inequality measures. This study quantifies maternal health inequalities across 31 Chinese provinces and identifies key socioeconomic and health system determinants.

**Methods:**

Using provincial panel data (2009–2021), we measured absolute and relative MMR disparities via the Slope Index of Inequality (SII) and Relative Index of Inequality (RII). Random-effects meta-regression models assessed how socioeconomic (population size, urbanization, college student numbers, total wealth, GDP per capita, insurance coverage) and health system variables (health workforce, beds, health expenditures per capita) influenced inequality, with stratification by eastern vs. central/western regions.

**Results:**

SII and RII varied considerably across provinces, indicating significant heterogeneity. Total wealth and higher education levels consistently reduced inequality, especially in central/western regions. In contrast, larger population size, urbanization, and uneven workforce distribution were associated with greater disparities. Health insurance had a protective effect mainly in less-developed areas. Paradoxically, higher health workforce density and rapid urbanization correlated with increased inequality, suggesting structural inefficiencies and unequal resource concentration.

**Conclusion:**

Household income, education, and insurance coverage are crucial for reducing maternal health disparities, whereas uneven urbanization and workforce allocation exacerbate them. Region-specific governance—focusing on fiscal transfers, education investment, and equitable health workforce distribution in central/western provinces, alongside efficiency improvements in the east—is essential for achieving Healthy China 2030 and SDG 3.1. Future policies must prioritize equity alongside average improvements.

## 1 Introduction

Maternal health is a cornerstone of women’s and children’s health and a critical indicator of social development and the performance of health systems. Since the launch of the Healthy China 2030 strategy, China has made remarkable progress in reducing maternal mortality, with maternal mortality ratios (MMR) in some regions approaching the levels of upper middle–income countries. However, substantial inequities remain. Significant provincial disparities persist, closely linked to socioeconomic development, the distribution of health resources, urbanization, insurance coverage, and educational attainment. These disparities suggest that while overall maternal health outcomes have improved, vulnerable populations and disadvantaged regions have not benefited equally, raising concerns of fairness and social justice (1, 2). At the global level, the World Health Organization (WHO) has emphasized that reducing inequities in maternal health is central to achieving the Sustainable Development Goal (SDG) target 3.1, lowering the global MMR to fewer than 70 per 100,000 live births by 2030. For a vast and socioeconomically diverse country such as China, understanding and addressing inter-provincial disparities in maternal health is therefore of particular importance (3, 4). While it is clear that both socioeconomic factors and health system factors play a crucial role in shaping maternal mortality outcomes, understanding the intricate relationships between these factors requires a deeper examination of existing research. The following literature review delves into how socioeconomic conditions and health system components interact to influence maternal health outcomes, shedding light on regional and demographic disparities that remain a significant challenge to reducing maternal mortality.

### 1.1 Determinants of socioeconomic factors influencing maternal mortality rate (MMR)

A substantial body of existing research has highlighted the crucial role of socioeconomic conditions in shaping maternal health outcomes. Factors such as per capita GDP, household income, and levels of urbanization have been shown to directly affect both the accessibility and quality of maternal health services. Wealthier regions typically experience lower maternal mortality, as economic prosperity often leads to improved healthcare infrastructure and greater access to medical services (1, 2). Higher income levels enhance the ability to afford essential healthcare, while urbanization provides easier access to medical facilities equipped with better resources. Empirical data from high-income countries, including Australia and Canada, indicate that the centralization of services in urban areas, coupled with inadequate primary care infrastructure, may diminish healthcare accessibility for populations residing in rural and peri-urban regions, notwithstanding the general economic affluence of these settings.

In addition to income and urbanization, education levels also play a pivotal role in maternal health. A higher level of schooling is strongly associated with a reduced risk of maternal mortality, as it enhances women’s ability to make informed decisions about their health and healthcare options (5, 6). Furthermore, health insurance coverage is another significant factor. In this study, the term ‘health insurance’ denotes China’s fundamental social medical insurance programs, excluding private insurance schemes. Aligning with international research, universal health coverage offers more robust protection against inequality compared to private insurance models. Broader insurance coverage is particularly important for improving service utilization and offering financial protection, particularly in rural areas where access to healthcare may otherwise be limited. In western China, for instance, extending insurance coverage has been shown to increase service utilization and provide vital financial protection against the high costs of maternal healthcare (4, 6).

However, social stratification and gender inequality continue to disadvantage vulnerable groups, even when healthcare services are theoretically accessible. These factors contribute to compounded inequalities in both the access to and utilization of services, which are particularly pronounced in underserved and remote regions. People in such areas often face greater challenges in obtaining care, despite the availability of medical services, which exacerbates maternal health disparities (4, 7, 8).

Global projections indicate that maternal mortality will persist at high levels in certain subgroups and regions unless substantial investments are made to address these inequalities, aligning with the World Health Organization’s (WHO) and the Sustainable Development Goals (SDG) 3.1 priorities, which aim to reduce maternal mortality worldwide (9, 10).

### 1.2 Determinants of health system factors influencing maternal mortality rate (MMR)

Alongside socioeconomic determinants, the health system plays a critical role in reducing maternal mortality. Global research indicates that transitioning from hospital-centered frameworks to primary health care and integrated, comprehensive models enhances accessibility, continuity of care, and public confidence in the health system. These factors serve as critical mechanisms for improving maternal health outcomes and promoting equity. The availability, quality, and geographic accessibility of healthcare resources are key elements that determine maternal health outcomes. A critical factor in improving maternal safety is the presence of adequately trained healthcare professionals, particularly midwives, and an efficient referral system to manage complications during childbirth. In regions where there are sufficient healthcare workers and well-organized referral systems, the maternal mortality rate is generally lower (3, 4, 11).

Geographical location is an important social determinant of maternal health inequality. Thaddeus and Maine (12), in their “three delays model,” pointed out that geographic barriers significantly delay pregnant women’s access to emergency obstetric care and constitute one of the key causes of maternal death. Gabrysch and Campbell (13) further argued that the spatial accessibility of medical services and road conditions directly determine whether pregnant women can receive timely treatment within the critical two-hour window, while rural and remote areas often suffer from “geographical health disadvantages” due to poor transportation and scarce facilities. The global study by Say et al. (14) revealed that maternal mortality rates in geographically isolated areas are two to three times higher than those in urban regions. Domestic scholars such as Zhang Lili et al. (15) found through interprovincial comparisons that maternal mortality rates in the western high-altitude and mountainous regions of China are significantly higher than in the eastern coastal areas, largely due to imbalances in transportation, economy, and medical resources. Liu Xiaohong (16) also noted that geographical location reinforces regional disparities in maternal health by affecting medical accessibility and resource allocation. Thus, geography is not merely a matter of physical distance but more profoundly reflects the structural inequality underlying regional development and health equity.

Finally, health financing and financial protection are crucial to achieving equitable healthcare access. A high proportion of out-of-pocket (OOP) payments forces many women to forgo necessary medical care due to cost, which contributes to higher risks during childbirth and worsens existing inequalities. In contrast, increasing government health expenditure and expanding insurance coverage are proven strategies to improve access to maternal healthcare, increase facility-based delivery rates, and reduce catastrophic health expenditures. Progress toward universal health coverage is critical in reducing maternal mortality by making healthcare more accessible and affordable to all (7, 8, 17, 18).

Nevertheless, three key limitations remain in the current literature. First, most studies focus on individuals or households, leaving a gap in systematic provincial-level analyses that could explain regional heterogeneity. Second, although international meta-analyses exist, they typically rely on cross-country comparisons and fail to reflect the unique administrative and socioeconomic characteristics of Chinese provinces. Third, much of the research emphasizes reducing average maternal mortality while neglecting equity, overlooking the fact that lowering overall mortality does not necessarily translate into fairer outcomes across social groups and may even mask growing disparities. Few studies have applied equity metrics such as the Slope Index of Inequality (SII) and the Relative Index of Inequality (RII), which are widely used in global health research to quantify disparities between advantaged and disadvantaged groups (19). Moreover, the pathways through which socioeconomic and health system factors contribute to maternal health inequities remain insufficiently explored, particularly in the context of China’s regional diversity.

To fill these gaps, this study uses provincial panel data from 2009 to 2021 across 31 provinces in China. By applying SII and RII to measure maternal health inequities and employing multivariate random-effects meta-regression, it systematically investigates how socioeconomic and health system factors shape patterns of maternal health disparities and how these mechanisms differ across regions with varying levels of development.

## 2 Materials and method

### 2.1 Data sources

#### 2.1.1 Data sources and classification

This study used data from the “China Health Statistics Yearbook” and “China Statistical Yearbook” of 31 provinces (municipalities or autonomous regions) from 2010 to 2022. In this study, the outcome variables were the SII and RII, while the socioeconomic and health system indicators listed below served as covariates.

The data on health resources and human resources, including outcome variables (institutions per 1,000 people (IPK), beds per 1,000 people (BPK), the numbers of doctors per 1,000 people (DPK), technicians per 1,000 people (TPK), nurses per 1,000 people (NPK), population size (PS), population density (PD), maternal mortality rate (MMR), per capita gross domestic product (PCGDP), urbanization level (UL), number of college students (NCS), numbers of insured (NI), per capita health expenses (PCHE), out-pocket payment (OPP), government health expenditures (GHE), geographical location (GL)). The variables and their descriptive characteristic were present in Table 1. We included 31 mainland provinces because Hong Kong, Macao, and Taiwan follow different maternal health reporting systems and lack comparable annual provincial data for 2009–2021.

**Table 1:** Variables and their description in the study.

Variables	Description	Unit	Data type	Data resource
Outcome variables				
SII	Slope Index of Inequality	None	Continuous	Calculated
RII	Relative Index of Inequality	None	Continuous	Calculated
Covariates				
MMR	The annual number of female deaths per 100,000 live births	Persons per 100,000 live births	Continuous	China Health Statistics Yearbook
IPK	Institutions per 1,000 people	None	Continuous	China Health Statistics Yearbook
BPK	Beds per 1,000 people	None	Continuous	China Health Statistics Yearbook
DPK	Doctors per 1,000 people	None	Continuous	China Health Statistics Yearbook
TPK	Technicians per 1,000 people	None	Continuous	China Health Statistics Yearbook
NPK	Nurses per 1,000 people	None	Continuous	China Health Statistics Yearbook
TW	Total wealth			
PS	Population size	None	Continuous	China Statistical Yearbook
UL	Urbanization Level	%	Continuous	China Statistical Yearbook
NFI	Net Farmers’ Income	Yuan	Continuous	China Statistical Yearbook
DIUR	Disposable Income of Urban Residents	Yuan	Continuous	China Statistical Yearbook
NCS	Number of College Students	None	Continuous	China Statistical Yearbook
NI	The number of Insured population	10,000 persons	Continuous	China Statistical Yearbook
PCHE	*Per Capita* Health Expenditures	None	Continuous	China Statistical Yearbook
OPP	Out-of-Pocket Expenditure on health	None	Continuous	China Statistical Yearbook
PCGDP	*Per Capita* Gross Domestic Product	Yuan	Continuous	China Statistical Yearbook
GHE	Annual Government Health Expenditures	None	Continuous	China Statistical Yearbook
GL	Geographical Location	None	Categorical	China Statistical Yearbook

SII, slope index of inequality; RII, relative index of inequality; IPK, institutions per 1,000 people; BPK, beds per 1,000 people; DPK, doctors per 1,000 people; TPK, technicians per 1,000 people; NPK, nurses per 1,000 people; PS, population size; PD, population density; MMR, maternal mortality rate; GHE, government health expenditures; OOP, Out-of-Pocket Payment; PCHE, per capita health expenditures; PCGDP, per capita gross domestic product; NI, number of insured; NCS, number of college students; DIUR, disposable income of urban residents; NFI, Net Farmers’ Income; GL, geographical location; UL, urbanization level.

Notedly, MMR is widely recognized indicators of healthcare needs in epidemiological and health policy literature (e.g., WHO benchmarks). It reflect population-level health risks that necessitate resource allocation (e.g., areas with high MMR require more maternal health services). The variable was selected to capture health needs distinct from socioeconomic status (SES), aligning with studies like Victora CG et al. (20) and Dong et al. (48), which use health outcomes to standardize need (21).

#### 2.1.2 Data processing

To facilitate meta-regression analysis and effectively manage the number of covariates, several variables were aggregated. Specifically, disposable income of urban residents (DRUI) and net farmers’ income (NFI) were combined to form a composite measure of total wealth (TW), calculated as the average of the two variables. Similarly, three categories of health human resources—namely, the number of doctors, technicians, and nurses per 1,000 population—were consolidated into a single indicator of healthcare workforce (HW), represented by the average of these three measures. Additionally, government health expenditures (GHE) and out-of-pocket health expenditures (OOP) were merged into an average health input (AHI) variable, computed as the mean of the two expenditure types.

### 2.2 Methods

#### 2.2.1 Slope index of inequality (SII) and relative index of inequality (RII)

To our best of knowledge, two regression-based measures—the slope index of inequality (SII) and its relative counterpart, the relative index of inequality (RII)—are frequently advocated (20) and employed for monitoring purposes, either independently or in conjunction with other indicators. The SII quantifies the gradient of health outcomes across multiple socio-economic (SE) groups that can be naturally ordered, achieved by rescaling the SE groups according to the relative position of each level. Conversely, the RII represents the ratio of health outcome levels between the theoretical lowest and highest positions within the SE hierarchy (21). These indices are purported to offer two principal advantages: (1) they incorporate information from all SE strata, and (2) they adjust for changes in the socio-economic composition of the population (22). Accordingly, we assessed both absolute and relative wealth-related inequalities in maternal mortality ratio (MMR) by employing the Slope Index of Inequality (SII) and the Relative Index of Inequality (RII) in the study, respectively. The Relative Index of Inequality (RII) is computed by ranking a weighted sample of the entire population from the most disadvantaged subgroup (assigned a rank of 0) to the most advantaged subgroup (assigned a rank of 1). This ranking incorporates weights that reflect the proportional distribution of the population across each subgroup. Subsequently, each subgroup’s population is represented by the midpoint of its range within the cumulative population distribution. Following the methodology implemented in the Health Equity Assessment Toolkit (HEAT) (23), the health indicator of interest is regressed against these midpoint values using a generalized linear model with a logit link function. Predicted values of the health indicator are then estimated at the two extremes of the distribution (ranks 0 and 1). The Equations (1-6) are presented below:

For health indicators where higher values denote favorable outcomes, the RII is calculated as the ratio of the predicted value at rank 1 (v₁) to that at rank 0 (v₀), encompassing the entire population distribution:


$$\mathrm{RII}=v_{1}/v_{0}$$ (1)

Conversely, for adverse health indicators, the RII is determined by the inverse ratio, that is, the predicted value at rank 0 divided by that at rank 1:


$$\mathrm{RII}=v_{0}/v_{1}$$ (2)

For favorable health indicators, values larger than one indicate a concentration of the indicator among the advantaged and values smaller than one indicate a concentration of the indicator among the disadvantaged. For adverse health indicators, values larger than one indicate a concentration of the indicator among the disadvantaged and values smaller than one indicate a concentration of the indicator among the advantaged.

SII represents the difference in estimated values of a health indicator between the most-advantaged and most-disadvantaged (or vice versa for adverse health indicators), while taking into consideration all the other subgroups – using an appropriate regression model.

To calculate SII, a weighted sample of the whole population is ranked from the most-disadvantaged subgroup (at rank 0) to the most-advantaged subgroup (at rank 1). This ranking is weighted, accounting for the proportional distribution of the population within each subgroup.

For favorable health indicators, the difference between the estimated values at rank 1 (𝑣1) and rank 0 (𝑣0) (covering the entire distribution) generates the SII value:


$$\mathrm{SII}=v_{1}-v_{0}$$ (3)

For adverse health indicators, the calculation is reversed and the SII value is calculated as the difference between the estimated values at rank 0 (𝑣0) and rank 1 (𝑣1) (covering the entire distribution):


$$\mathrm{SII}=v_{0}-v_{1}$$ (4)

For favorable health indicators, positive values indicate a concentration of the indicator among the advantaged and negative values indicate a concentration of the indicator among the disadvantaged. For adverse health indicators, positive values indicate a concentration of the indicator among the disadvantaged and negative values indicate a concentration of the indicator among the advantaged.

It is acknowledged that the calculation of the Slope Index of Inequality (SII) and the Relative Index of Inequality (RII) is optimally conducted using individual-level data or finely stratified data at a single time point. In the present study, however, we employed provincial-level time-series aggregate data as a pragmatic and methodologically established alternative. Specifically, for each province, thirteen annual observations spanning 2009 to 2021 were ranked and categorized into wealth quintiles based on a composite income indicator, thereby constructing a temporal socioeconomic gradient within each province. The mean maternal mortality ratio (MMR) was then computed for each quintile. The SII was estimated from the slope of the regression line relating the mean MMR values to the median wealth values corresponding to each quintile. This approach captures the ecological gradient at the provincial level over time, rather than individual-level inequality. Although this methodology does not directly measure inequality among individuals, it offers a valid assessment of the association between temporal changes in provincial wealth and maternal health outcomes. Such insights remain valuable for monitoring purposes and policy formulation.

#### 2.2.2 Meta regression analyses

Meta-analysis constitutes a rigorous methodological framework for integrating quantitative findings from multiple independent studies addressing a shared research question, with a principal aim of estimating aggregated effect sizes across these studies (24). In the present study, we applied random-effects meta-regression to examine the influence of socioeconomic and healthcare resource variables on regional disparities in maternal mortality ratio (MMR) inequalities. Meta-regression analysis, used to explore heterogeneity in meta-analysis, can be summarized with the formula:


θ^=β0+β₁X₁+…+βpXp+ε+ζ (5)

where θ^ is the estimated effect size, X₁,…, Xₚ are study-level predictors, *β*₀ is the intercept, β₁, …, βₚ are the regression coefficients, *ε* is the sampling error, and *ζ* represents between-study heterogeneity.

The random-effects meta-regression model in the study is formally expressed as:


θ^=β0+ΣjβjXji+ui+εi (6)

Where θ^ᵢ: The observed effect size (e.g., SII or RII) for province I;β₀: Intercept term representing the average effect across provinces; Xⱼᵢ: Matrix of j predictors for province i, including: socioeconomic variables: PS, TW, PCGDP, NCS, NI, UL etc., and health system and healthcare resource variables: AHI, PCHE, IPK, BPK, HW;βⱼ: Regression coefficients quantifying how predictors modify the baseline effect;uᵢ: Between-province random effect ∼ N(0,τ^2^);εᵢ: Within-province sampling error ∼ N(0,σᵢ^2^).

This sophisticated analytical technique offers three primary advantages. First, by synthesizing data derived from provincial-level investigations, it facilitates the quantification of how macro-level determinants—such as PCGDP, UL, and healthcare resource distribution—mediate the association between regional inequalities and maternal health outcomes. Second, the approach explicitly models heterogeneity across studies, thereby enhancing the reliability and validity of the results. Third, by identifying modifiable regional factors, it provides empirically grounded policy recommendations aimed at mitigating spatial health disparities.

The adoption of a random-effects model is particularly suitable for this context, as it accommodates inherent variability among provinces while simultaneously evaluating hypothesized predictors of MMR inequality patterns. This methodological strategy transcends conventional pooled effect estimates by elucidating contextual influences that may account for the differential manifestation of maternal health disparities across geographic areas.

We conducted multivariate meta-regression using restricted maximum likelihood (REML) estimation, with Knapp-Hartung adjustment for small-sample bias (25). For the sake of computational efficiency, this study employed a logarithmic transformation of the Relative Inequality Index (RII). Variables were incorporated into the meta-regression analysis if their variance inflation factors (VIFs) were below the threshold of 10 or if they exhibited statistical significance in relation to SII values in univariate analyses. Statistical significance was set at *p* < 0.05 (two-tailed), with exponentiated coefficients (exp(*β*)) reported for interpretability. Heterogeneity was quantified via I^2^ and τ^2^. Analyses were performed in Stata/MP 17.0 (StataCorp), using the metareg command with inverse-variance weighting. Bootstrap resampling (500 replicates) validated robustness.

## 3 Results

### 3.1 Results of descriptive statistics

As shown in Table 2, the distribution of provincial-level indicators for maternal mortality ratio (MMR) equity—namely the Slope Index of Inequality (SII, absolute disparity) and the Relative Index of Inequality (RII, relative disparity)—varied considerably across China. Only two provinces from 32 presented positive SII (Liaoning and Jiangsu), indicating that maternal health resources or outcomes tended to concentrate among advantaged groups, with pronounced interprovincial differences (e.g., Xizang with an SII as low as −0.0076 versus Beijing at only −0.00024). RII values exhibited an extremely wide range (0.00002182 to 118.06), with provinces such as Jiangsu and Liaoning recording exceptionally high levels, suggesting severe relative inequality. Almost half of provinces then fell below 1 (15/31), implying that maternal health outcomes disproportionately favored advantaged groups (According to the methods section presented above, the variable “advantaged” denotes province-year observations corresponding to higher wealth quintiles. This approach effectively captures the socioeconomic gradient at the provincial level across time and facilitates the estimation of inequality indices despite limitations in the available data). Only Shanghai and Liaoning showed maternal mortality rates concentrated among disadvantaged groups. In sum, more developed provinces revealed greater internal disparities, while less developed provinces exhibited higher overall risks but smaller within-province inequalities (A development ranking of provinces is provided in Supplementary Table S1). Table 2 also illustrates the provincial distribution of SII and RII.

**Table 2:** Provincial distribution of SII and RII for MMR across mainland China from 2009 to 2021.

Provinces	SII_matmor	RII_matmort
Beijing	−0.00024011	0.00010371
Tianjin	−0.00009903	0.08683872
Hebei	−0.00022063	0.01494837
Shanxi	−0.00029508	0.00615661
Neimenggu	−0.00077683	8.464e-08
Liaoning	0.00011941	11.256292
Jilin	−0.00093345	1.274e-07
Heilongjiang	−0.00011065	0.15443696
Shanghai	−0.00007486	0.05076122
Jiangsu	0.00017046	118.05552
Zhejiang	−0.00012042	0.01558178
Anhui	−0.00054186	9.228e-06
Fujian	−0.00022203	0.00478962
Jiangxi	−0.00037568	0.00042964
Shandong	−0.00018202	0.0183495
Henan	−0.00024903	0.01210764
Hubei	−0.00028625	0.00369384
Hunan	−0.00083484	1.494e-08
Guangdong	−0.00011327	0.06349546
Guangxi	−0.00075696	1.483e-06
Hainan	−0.00031963	0.00172982
Chongqing	−0.00084434	2.595e-08
Sichuan	−0.00067899	9.920e-07
Guizhou	−0.00138306	1.344e-11
Yunnan	−0.00156935	1.931e-13
Xizang	−0.00756345	0.00000
Shanxi	−0.00056648	0.00002182
Gansu	−0.00140824	9.941e-11
Qinghai	−0.00142748	1.134e-11
Ningxia	−0.00083451	3.607e-07
Xinjiang	−0.00140171	5.183e-12

SII, slope index of inequality; RII, relative index of inequality; MMR, maternal mortality rate.

Further statistical analysis (Table 3) indicated that the mean MMR across provinces was 17.85 deaths per 100,000 live births, but the maximum reached 118.49, highlighting enormous regional variation. The mean SII (−0.00078) was negative, reaffirming the unexpected concentration of maternal mortality risk among advantaged populations. In this study, negative SII values indicate that higher maternal mortality risk is concentrated among the advantaged temporal wealth groups. This interpretation aligns with our constructed temporal ranking and differs from conventional group-based inequality interpretation. We have now clarified this in Section 2.2. The mean RII was 4.185, with a maximum of 118.06, implying that maternal health outcomes disproportionately favored disadvantaged groups. While health resources such as beds per 1,000 people (BPK) and healthcare workforce (HW), as well as average health input (AHI), were relatively evenly distributed, numbers of insured (NI) and per capita gross domestic product (PCGDP) varied sharply, suggesting that economic development and health financing imbalances may be important contextual factors behind maternal health inequities.

**Table 3:** Describtive analysis of the variables of the interest in the study.

Variables	Mean	SD	Min	p50	Max	N
SII	−0.0007787	0.001351	−0.008	−0.0003757	0.0001705	31
RII	4.185	21.230	0.000	0.0001037	118.056	31
PS	4420.712	2846.758	326.923	3817.692	11055.923	31
TW	21203.771	5941.660	15247.427	18952.962	39161.900	31
MMR	17.852	19.967	4.085	13.533	118.492	31
IPK	0.771	0.356	0.262	0.725	1.879	31
BPK	5.128	0.623	3.978	5.180	6.393	31
HW	3.652	0.662	2.760	3.499	6.407	31
AHI	535.300	251.311	110.187	485.052	1208.342	31
PCHE	3413.068	1366.653	2325.060	3144.070	8852.570	31
NI	2739.546	1955.958	142.969	2243.723	9084.431	31
NCS	3170.918	1095.334	1905.769	2876.077	7403.923	31
PCGDP	53249.652	23814.482	26324.438	43306.962	116000.000	31
GL	2.032	0.875	1.000	2.000	3.000	31
UL	56.964	12.656	30.690	54.983	88.831	31

### 3.2 Results of univariate analysis

Table 4 presents the univariate meta-regression estimates of the associations between provincial socioeconomic and health system indicators and average maternal mortality, with stratification by region (loca = 1 likely denoting eastern provinces, loca = 3 central and western provinces). Because the dependent variable in this section is MMR itself, the estimates reflect determinants of mortality levels rather than determinants of inequality, distinguishing this analysis from Section 3.3 where SII and RII serve as outcomes. Instead, Table 4 provides necessary contextual understanding of how provincial characteristics relate to overall mortality levels.

**Table 4:** Univariate meta-regression estimates for the association between province-level socioeconomic and health system indicators and mean MMR.

Variables	Total population (*N* = 31)		Eastern region = 1 (*N* = 11)		Western region = 3 (*N* = 20)	
	β (95% CI)	Adj R^2^	β (95% CI)	Adj R^2^	β (95% CI)	Adj R^2^
PS	−0.00050 (−0.00118, 0.00019)	18.2%	0.00001 (−0.00061, 0.00062)	9.5%	−0.00095 (−0.00188, −0.00002)**	39.12%
TW	−0.00050 (−0.00068, −0.00032)***	92.3%	−0.00035 (−0.00061, −0.00009)**	91.6%	−0.00221 (−0.00424, −0.00019)**	97.8%
IPK	4.94850 (−0.78595, 10.68295)*	19.00%	3.47047 (−1.27221, 8.21315)	73.36%	2.12153 (−1.1e+01, 15.14463)	39.12%
BPK	1.77830 (−1.46116, 5.01775)	6.5%	−0.56222 (−3.56024, 2.43579)	3.1%	4.54115 (0.19004, 8.89226)**	90.20%
HW	−2.49190 (−5.41983, 0.43604)*	23.56%	−2.04026 (−4.96099, 0.88047)	8.9%	2.82236 (−3.45218, 9.09690)	15.44%
AHI	−0.00608 (−0.01373, 0.00156)	10.2%	−0.00034 (−0.00723, 0.00655)	6.4%	−0.00892 (−0.02100, 0.00315)	14.3%
PCHE	−0.00137 (−0.00259, −0.00016)**	43.92%	−0.00091 (−0.00202, 0.00021)	93.92%	0.00657 (0.00145, 0.01168)**	90.8%
NI	−0.00082 (−0.00175, 0.00010)*	7.9%	−0.00005 (−0.00087, 0.00078)	3.8%	−0.00167 (−0.00330, −0.00005)**	41.19%
NCS	−0.00202 (−0.00368, −0.00035)**	16.46%	−0.00060 (−0.00218, 0.00099)	24.3%	−0.00488 (−0.00998, 0.00022)*	40.7%
PCGDP	−0.00012 (−0.00017, −0.00007)***	89.41%	−0.00008 (−0.00015, −0.00001)**	91.37%	−0.00013 (−0.00050, 0.00024)	12.1%
UL	−0.22566 (−0.35603, −0.09529)***	48.02%	−0.10937 (−0.26392, 0.04518)	13.94%	−0.03568 (−0.55726, 0.48590)	26.3%

Analysis revealed that both total wealth (TW) and PCGDP showed highly significant negative associations with MMR across the full sample and in both subgroups (coefficients consistently negative, with confidence intervals not overlapping zero). The adjusted R^2^ values were extremely high (92.3 and 89.4% in the full sample), indicating that economic strength is the most critical determinant of reducing maternal mortality, explaining the majority of observed variation. This pattern reflects the broad role of economic development in shaping population health, but should not be interpreted as evidence that these variables reduce wealth-related inequality, which is examined separately in Section 3.3.

Regional stratification further indicated differential mechanisms. For example, in central and western provinces, the accessibility of basic medical hardware was particularly important. The number of BPK exhibited a significant positive association (*β* = 4.54, *p* < 0.01, Adj R^2^ = 90.2%), suggesting that in resource-limited central and western regions, increasing facility supply alone may paradoxically worsen maternal mortality outcomes, possibly due to inefficiencies in utilization. This finding should not be interpreted as indicating worsening inequality; instead, it suggests that expanding physical infrastructure in resource-constrained regions does not immediately translate into reduced mortality, possibly due to limitations in staffing, referral capacity, or service quality. While in eastern provinces, BPK showed insignificant negative relationship (Adj R^2^ = 3.1%, *p* > 0.05).

Similarly, insurance coverage also showed regionally heterogeneous effects: in central and western provinces, higher NI was significantly associated with reduced maternal mortality, while it does not work at all in the eastern region, though it remained negative significant association with MMR in the full sample. It was also for the case of number of college students (NCS). Notedly, a comparable pattern appeared for per capita health expenses (PCHE), which was negatively associated with MMR nationwide but positively in central and western regions, implying reverse causality and inefficient expenditure allocation in less developed areas. By contrast, PCGDP remained negatively and significantly associated with MMR in both the full sample and eastern provinces, reflecting the strong protective effect of economic development; however, its insignificance in central and western regions suggests that the benefits of growth have not been effectively translated into maternal health gains due to weaker fiscal capacity and limited service accessibility. These findings underline the critical role of region-targeted financial protection and government health investment. Only one socioeconomic factor—TW, the average of urban and rural residents’ income—emerged as a consistently significant driver of maternal health outcomes across all models. It indicated that overall household income level, rather than aggregate economic scale, serves as the most direct and equitable determinant of maternal survival, reflecting how income growth enhances care affordability, nutrition, and timely access to obstetric services.

Importantly, these univariate relationships describe mortality-level associations only. They do not reflect whether socioeconomic or health system variables widen or narrow inequality, which is the focus of the meta-regression models in Section 3.3.

**Figure 1:**
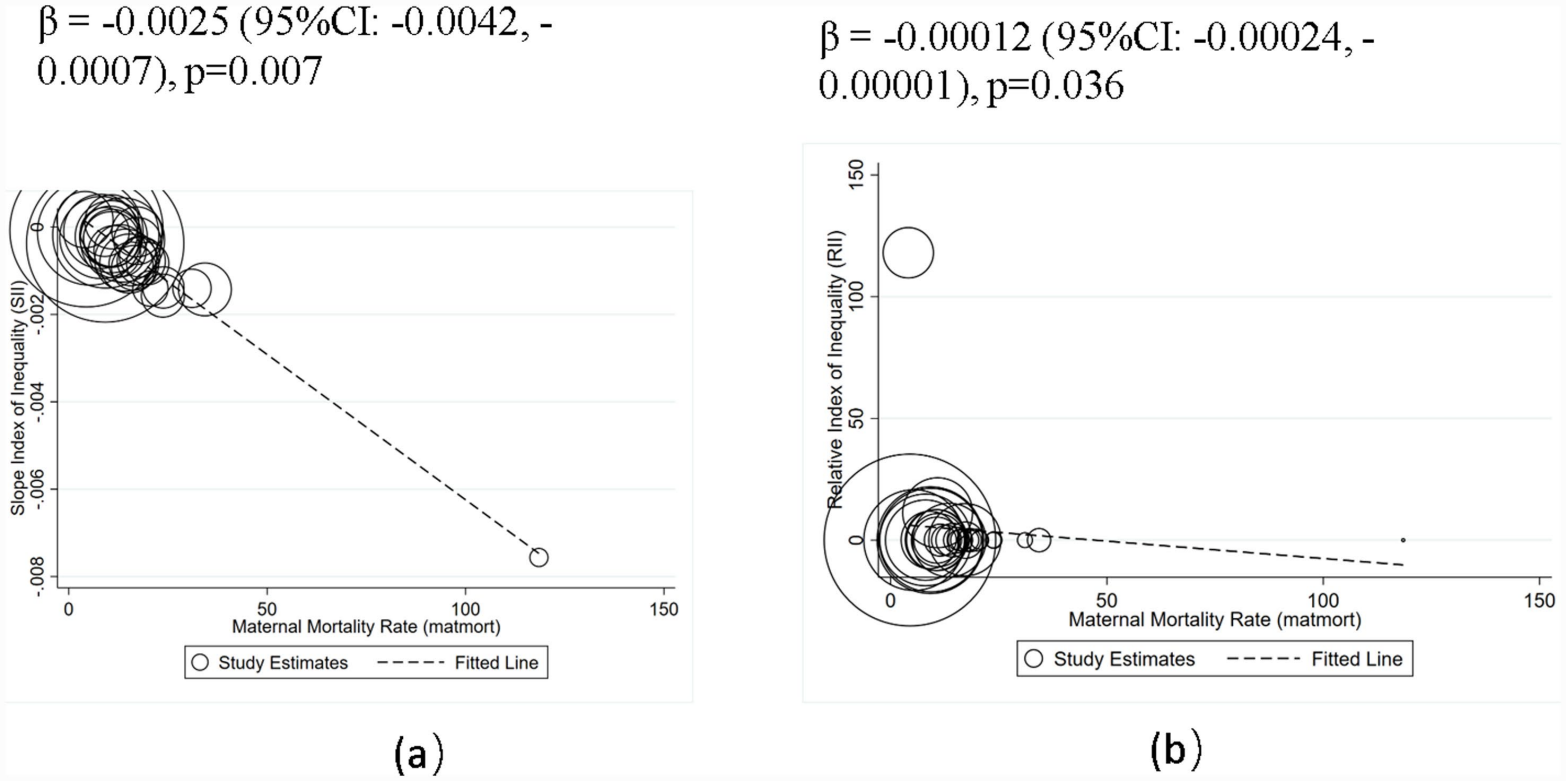
Negative RIIs and SIIs indicate pro-rich inequality in MMR. Bubble size proportional to the precision (inverse variance) of country-specific estimates.

A bubble plot analysis further demonstrated a strong, significant negative relationship between maternal mortality and health inequality. Absolute inequality (SII) was more strongly and negatively associated with MMR (larger negative values), while relative inequality (RII) showed a weaker negative association (smaller positive values). This indicates that higher maternal mortality correlates with greater absolute inequality but lower relative inequality (Figure 1).

### 3.3 Results of meta-regression analysis

Table 5 presents the results of multivariate meta-regression analyses investigating the relationships between socioeconomic and health system factors and wealth-related disparities in maternal mortality, as measured by the SII and the logarithm of the Relative Index of Inequality (logRII). Given that the dependent variables in these models represent inequality indices rather than absolute MMR, the interpretations herein pertain specifically to the effects of the predictors on the distribution of maternal mortality along the temporal wealth gradient within provinces, rather than on the aggregate mortality rates.

**Table 5:** Meta-regression estimates for the association between MMR inequality and province-level determinants.

Country-level predictor	National (*N* = 31)				Eastern region (*N* = 11)		Central and Western region (*N* = 20)	
	Multivariate		Adjusted for region fixed effects		Multivariate		Univariate	
	SII (95% CI)	logRII (95% CI)	SII (95% CI)	logRII (95% CI)	SII (95% CI)	logRII (95% CI)	SII (95% CI)	logRII (95% CI)
PS	1.55e-07**	0.00298*	1.20e-07*	0.00252*	−5.68e-08	−0.00167	2.26e-07*	0.00514**
	(1.40e-08–2.96e-07)	(0.000149–0.00581)	(−3.19e-09–2.43e-07)	(3.75e-05–0.00501)	(−4.51e-07–3.38e-07)	(−0.0119–0.00860)	(4.54e-08–4.08e-07)	(0.00179–0.00849)
TW	−5.52e-08***	−0.00110***	−5.92e-08***	−0.00128***	−7.18e-08	−0.00159	−1.69e-07**	−0.00386***
	(−8.22e-08– −2.81e-08)	(−0.00165– −0.000546)	(−8.28e-08– −3.57e-08)	(−0.00177– −0.000784)	(−2.19e-07–7.59e-08)	(−0.00527–0.00209)	(−2.47e-07– −9.06e-08)	(−0.00536– −0.00235)
MMR	−5.84e-05***	−1.000***	−5.61e-05***	−1.016***	−8.65e-05	−2.055	−5.17e-05***	−0.841***
	(−7.05e-05– −4.63e-05)	(−1.128– −0.872)	(−6.74e-05– −4.47e-05)	(−1.124– −0.907)	(−0.000235–6.24e-05)	(−5.923–1.813)	(−6.50e-05– −3.84e-05)	(−0.968– −0.713)
HW	0.000231**	2.969	0.000289**	4.788**	0.000356	5.553	−1.53e-05	0.254
	(8.93e-06–0.000453)	(−1.456–7.394)	(9.67e-05–0.000482)	(0.830–8.745)	(−0.000806–0.00152)	(−23.43–34.54)	(−0.000281–0.000250)	(−4.821–5.329)
NI	−1.87e-07*	−0.00362*	−1.59e-07	−0.00340*	3.86e-08	0.00131	−2.22e-07	−0.00539*
	(−3.78e-07– −1.26e-09)	(−0.00748– −0.000236)	(−3.24e-07–5.93e-09)	(−0.00675– −4.63e-05)	(−4.26e-07–5.04e-07)	(−0.0107–0.0133)	(−5.29e-07–8.58e-08)	(−0.0110– −0.000224)
NCS	−2.63e-07***	−0.00477**	−2.53e-07**	−0.00493**	−2.31e-07	−0.00409	−2.72e-07**	−0.00436**
	(−4.20e-07– −1.06e-07)	(−0.00787– −0.00166)	(−3.89e-07– −1.18e-07)	(−0.00762– −0.00225)	(−1.01e-06–5.47e-07)	(−0.0234–0.0153)	(−4.55e-07– −8.90e-08)	(−0.00738– −0.00134)
UL	4.54e-05***	0.898***	3.53e-05***	0.713***	2.25e-05	0.394	7.65e-05***	1.485***
	(2.58e-05–6.50e-05)	(0.496–1.299)	(1.78e-05–5.28e-05)	(0.354–1.072)	(−5.20e-05–9.70e-05)	(−1.437–2.224)	(5.12e-05–0.000102)	(0.992–1.978)
Constant	−0.00133**	−23.62*	−0.000687	−9.757	0.000658	28.34	−0.000361	−4.159
	(−0.00235– −0.000306)	(−43.65– −3.600)	(−0.00166–0.000282)	(−28.82–9.309)	(−0.00383–0.00515)	(−87.33–144.0)	(−0.00184–0.00111)	(−27.94–19.62)
Adj R2	97.64%	97.47%	98.46%	98.20%	7.30%	19.38%	99.45%	99.19%
I2_res	86.14%	100.00%	79.67%	100.00%	84.98%	100.00%	63.65%	100.00%
tau2	2.8e-08	14.25	1.8e-08	10.11	1.5e-08	11.99	1.0e-08	6.202
p	<0.001	<0.001	<0.001	<0.001	0.5011	0.4411	<0.001	<0.00120
Observation	31	31	31	31	11	11	20	

Seen from Table 5, population size (PS) showed a significant positive correlation with both SII and RII. In the full sample, SII: *β* = 1.55e-07 (*p* < 0.01); after controlling for regional fixed effects, the effect was slightly attenuated but remained significant (*β* = 1.20e-07, *p* < 0.05). In central and western provinces, the effect was even stronger (*β* = 2.26e-07, *p* < 0.05), while in eastern provinces the association was not significant. Similar patterns were observed for logRII: in the full sample: *β* = 0.00298 (*p* < 0.05); after controlling for fixed effects: *β* = 0.00252 (*p* < 0.05); in the central/west regions: *β* = 0.00514 (*p* < 0.01). Given that SII values are predominantly negative across provinces, these positive coefficients indicate an expansion of inequality in absolute terms only when SII is positive, but in most cases reflect movements of SII toward zero, that is, changes in the magnitude of inequality rather than increases in mortality itself.

TW was significantly negatively correlated with both inequality indices. In the full sample, SII: *β* = −5.52e-08 (*p* < 0.001); logRII: *β* = −0.00110 (*p* < 0.001). These results remained robust after controlling for regional fixed effects, and remained significant in the central and western subsample (SII: *β* = −1.69e-07, *p* < 0.01; logRII: *β* = −0.00386, *p* < 0.001), but not in the east. This highlights that overall household income, especially in less-developed regions, is a fundamental force in mitigating maternal health inequities. This indicates that higher household income is associated with a reduction in wealth-related inequality, especially in less-developed regions. Importantly, this finding should be interpreted strictly as reduced inequality, not as a reduction in absolute MMR, which is examined separately in Section 3.2.

MMR itself exhibited a strong negative correlation with inequality indices. In the full sample, SII: *β* = −5.84e-05 (*p* < 0.001); logRII: *β* = −1.000 (p < 0.001). After controlling for fixed effects, the results remained stable (SII: *β* = −5.61e-05, *p* < 0.001; logRII: *β* = −1.016, *p* < 0.001). Subgroup analysis revealed significance only in central/west provinces. This pattern is consistent with a “floor effect,” whereby provinces with uniformly high mortality levels display compressed inequality because risks are similarly elevated across wealth strata. This association reflects inequality dynamics rather than determinants of overall mortality.

The number of HW was positively associated with both inequality indices in the full sample, after controlling for regional effects (SII: *β* = 0.000289, *p* < 0.01; logRII: *β* = 4.788, *p* < 0.01). Given the predominantly negative SII values, this positive association does not imply a worsening of maternal mortality outcomes. Instead, it indicates a shift in the magnitude of inequality, potentially reflecting uneven distribution of workforce resources across the wealth gradient. However, in subgroup models, coefficients were not significant, suggesting that regional stratification already absorbed much of the between-region variation.

NCS was consistently negatively associated with both absolute and relative inequalities. In the full sample, SII: *β* = −2.63e-07 (*p* < 0.001); after controlling for fixed effects, *β* = −2.53e-07 (*p* < 0.01). Subgroup analysis showed significance in central/west provinces but not in the east. Similarly, for logRII, higher educational attainment reduced maternal health inequalities primarily in less-developed regions.

NI showed mixed effects. In the full sample, both SII and RII were significantly negatively associated (SII: *β* = −1.87e-07, *p* < 0.05; logRII: *β* = −0.00362, *p* < 0.05). After controlling for regional fixed effects, the SII association weakened (*β* = −1.59e-07, not significant), but logRII remained significant (*β* = −0.00340, *p* < 0.05). In the central/west model, insurance coverage remained weakly negatively associated with logRII (*β* = −0.00539, *p* < 0.05). In the east, associations were not significant. These results suggest a modest role of insurance coverage in reducing relative inequality, particularly in less-developed regions.

Urbanization level (UL) was positively associated with both inequality indices across all models. In the full sample, SII: *β* = 4.54e-05 (*p* < 0.001); logRII: *β* = 0.898 (*p* < 0.001). After controlling for fixed effects, associations remained significant (SII: *β* = 3.53e-05, *p* < 0.001; logRII: *β* = 0.713, *p* < 0.001). Subgroup analyses confirmed that urbanization significantly contributed to greater inequities in central/west provinces. Given the negative baseline SII in most provinces, these findings indicate that rapid urbanization alters the magnitude and structure of inequality rather than uniformly increasing mortality, suggesting that urban expansion without parallel improvements in primary care and service integration may exacerbate disparities in maternal health.

Notably, the explanatory power of models varied by region. In central and western provinces, adjusted R^2^ exceeded 98%, indicating strong explanatory strength. In contrast, eastern models explained only 7.3–19.4% of variance, with most predictors nonsignificant, suggesting that unobserved contextual factors may drive maternal health inequalities in wealthier provinces.

## 4 Discussion

This study demonstrates substantial regional disparities in maternal health equity in China. SII results indicated an unexpected concentration of mortality risk among advantaged groups, with marked interprovincial variation. RII values highlighted that within-province inequalities were more pronounced in economically developed provinces, while underdeveloped provinces faced higher overall risks but smaller internal disparities. Furthermore, MMR showed significant imbalance across provinces, and its negative correlation with inequality indices suggests that higher mortality is linked to greater absolute inequity but lower relative inequity.

To avoid conflation between mortality levels and inequality, it is important to note that the determinants of mean MMR (Section 3.2) are not necessarily the same as the determinants of wealth-related inequality (Section 3.3). Univariate analyses describe how provincial characteristics correlate with mortality levels, whereas the meta-regression models evaluate how these same characteristics shape disparities across the temporal wealth distribution. Consequently, factors such as total wealth, education, and insurance coverage may have strong effects on inequality even when their association with mean MMR differs. This analytical distinction clarifies that improving average maternal mortality and reducing inequality are related but conceptually separate policy objectives.

Although healthcare resources such as hospital beds and workforce distribution appeared relatively balanced, significant gaps in economic development and insurance coverage persist, providing important background conditions for inequity. Meta-regression analyses revealed pronounced regional heterogeneity: PS, UL, and unequal HW allocation intensified inequities, particularly in central and western regions. By contrast, overall household income, educational attainment, and expanded insurance coverage were strongly associated with reduced inequities, with more robust effects observed in less-developed regions than in the east, where saturation effects—i.e., diminishing returns to additional inputs once resource levels are already high—along with unmeasured contextual drivers, appear to dominate.

### 4.1 Positive associations with inequities

This study revealed that population size, urbanization, and health workforce levels were positively associated with inequities in maternal health outcomes. Importantly, given that SII values are predominantly negative across provinces, these positive coefficients should be interpreted as changes in the magnitude of inequality (often movements toward zero) rather than as worsening maternal mortality outcomes. These findings indicate that structural and demographic transformations—while often associated with progress—can inadvertently exacerbate disparities in healthcare access and quality if not managed through equity-oriented governance.

In central and western provinces, rapid population growth increased demands on already limited healthcare infrastructures, straining the capacity of local health systems and leading to uneven resource allocation. This aligns with previous evidence from Gabrysch and Campbell (13) and Ouma et al. (26), who emphasized that population density interacts with geographical constraints to deepen maternal health inequalities in resource-limited settings. More recent subnational analyses from Zhao et al. (27) and Zeng et al. (28) similarly observed that population expansion in underdeveloped western provinces of China amplified the service gap in emergency obstetric care, especially where fiscal decentralization restricted public investment in health infrastructure.

The role of urbanization in widening inequities reflects what scholars describe as the “urban health penalty” (The urban health penalty describes circumstances in which rapid urbanization leads to overcrowding, environmental stressors, or fragmented primary care systems that disproportionately affect vulnerable groups, explaining why higher urbanization levels in some provinces may correlate with widening maternal health disparities). While urban expansion enhances overall healthcare coverage, it can intensify spatial and social segregation within cities. Our results corroborate existing research indicating that rapid urbanization does not inherently lead to a reduction in inequality. In the absence of concurrent investments in primary healthcare, migrant-inclusive services, and integrated referral systems, disparities may intensify, especially among rural-to-urban migrants, residents of older urban villages, and peri-urban communities. Urban elites and insured formal-sector workers often access high-quality tertiary hospitals, whereas rural migrants and informal workers face barriers to institutional delivery due to household registration restrictions and limited insurance portability (29–31). Chen et al. (31) found that in rapidly urbanizing provinces, the maternal mortality rate among migrant women was up to 40% higher than that of registered urban residents, echoing the intra-urban disparities documented in Sub-Saharan Africa and Latin America (32). Consequently, strengthening primary healthcare, integrating maternal and child health services, and moving away from hospital-centric care models are critical strategies for mitigating maternal health inequalities. International evidence indicates that comprehensive, continuity-based care models—rooted in robust primary healthcare networks—can enhance trust, improve accessibility, and facilitate early detection of complications, thereby reducing disparities (33, 34).

The positive association between health workforce density and inequity underscores a distributional paradox. Although expanding the total number of health professionals is generally beneficial, uneven spatial allocation can aggravate inequalities. Skilled birth attendants and obstetricians remain concentrated in economically advantaged and urbanized regions, leaving peripheral communities underserved. This phenomenon, widely documented by Campbell and Graham (35), Homer et al. (11) persists even under national workforce expansion programs. Newer evidence from Liu et al. (32) and Zhang et al. (36) shows that China’s rapid growth in obstetric workforce density from 201 to 2018 disproportionately benefited tertiary hospitals in coastal regions, while primary maternal care institutions in rural western counties saw little change. Similarly, Shengelia et al. (37) highlighted that inequality in human resource distribution—especially midwives—was one of the strongest predictors of subnational maternal mortality variation across 74 low- and middle-income countries. Taken together, these findings support the conclusion that development without equity mechanisms can produce unintended stratification effects. Growth in population, infrastructure, or human resources will not automatically translate into equitable outcomes unless accompanied by redistributive policies ensuring balanced allocation and governance accountability.

### 4.2 Negative associations with inequities

In contrast, overall household income was negatively correlated with maternal health inequities, underscoring the pivotal role of economic development in improving equity in maternal outcomes. Rising income enhances household capacity to afford essential maternity care, including antenatal visits, skilled delivery, and timely emergency treatment, thereby reducing avoidable delays in the “three delays” framework. Higher income also alleviates financial barriers—such as user fees and transport costs—while mitigating the risk of catastrophic health expenditures. At the macro level, income growth expands fiscal capacity and social insurance contributions, enabling greater investment in obstetric infrastructure, referral systems, and the distribution of qualified medical personnel to underserved areas. This dual effect—improving both household affordability and system readiness—translates economic progress into measurable equity gains. Consistent with Marmot (7)and Gakidou et al. (6), recent studies by Ronsmans and Graham (38) and Bicaba et al. (39) confirm that regions with higher per capita GDP exhibit lower maternal mortality and narrower regional inequality gaps, largely through strengthened health financing and social protection systems. In this study, provinces with higher average resident income showed a reduced concentration of maternal deaths among poorer regions, indicating that income growth not only improves individual access to care but also narrows interprovincial disparities. Broadly, economic development functions as both a structural and behavioral equalizer, associating increased income levels with more equitable maternal health outcomes. Nonetheless, gross domestic product (GDP), as a macroeconomic measure, frequently conceals significant intra-provincial inequalities. Consequently, provinces categorized as economically prosperous may still encompass marginalized rural or mountainous communities that encounter considerable obstacles in accessing maternal health services.

Interestingly, the negative association between MMR and inequality suggests a “floor effect”—when maternal risks are universally high, interregional gaps shrink because mortality is elevated across all populations (The floor effect is explained in relation to our inequality metrics (SII/RII), highlighting how provinces with uniformly high MMR levels may display artificially compressed inequality simply because the overall level leaves “little room” for differential distribution). However, as overall MMR declines, disparities tend to widen until universal coverage and quality improvement catch up. This nonlinear pattern mirrors the “inequality paradox” discussed by Kruk et al. (40) and empirically supported by Afulani et al. (41), who showed that quality improvements often initially benefit better-off populations before diffusing to marginalized groups.

Education emerged as a consistently protective factor, narrowing inequities by improving health literacy, decision-making autonomy, and utilization of maternal services. Bloom et al. (5), Gakidou et al. (6), and Zhang et al. (42) all highlighted that education reduces preventable maternal deaths by empowering women to seek antenatal care and skilled attendance at birth. In recent studies, Wang et al. (43) and Yaya et al. (44) demonstrated that secondary and tertiary education are the strongest predictors of institutional delivery among poor women in both China and Sub-Saharan Africa, suggesting that educational parity can substantially reduce intergroup disparities in maternal outcomes.

Insurance coverage was another robust determinant of equity. The expansion of basic health insurance in central and western regions substantially reduced financial barriers to accessing obstetric care. This finding aligns with Garcia and Smith (17) and Wagstaff et al. (45), who demonstrated that lowering out-of-pocket (OOP) expenditures reduces catastrophic health spending and enhances equity in service use. Recent evaluations of China’s Urban–Rural Resident Medical Insurance Integration (URRMI) reform (2016–2020) confirm that merging fragmented schemes narrowed rural–urban inequities in maternal service utilization by improving benefit portability and pooling risks (46, 47). Similarly, WHO (9) and UNICEF & WHO (10) reaffirmed that achieving Universal Health Coverage (UHC) is the most effective strategy to eliminate maternal health inequities globally.

Overall, the results therefore emphasize that reductions in maternal mortality do not automatically translate into reductions in inequality, and vice versa. Interventions must simultaneously address both dimensions to achieve equitable maternal health improvement. These findings not only corroborate but extend previous research by highlighting the interactive nature of structural and policy variables. Economic development and social protection are most effective when coupled with equitable distribution of human resources, quality assurance mechanisms, and targeted support for marginalized populations. Sustainable reductions in maternal health inequity therefore require not only higher income and coverage rates but also institutional reforms that integrate spatial, fiscal, and social equity dimensions—a goal central to achieving SDG 3.1.

This study has also several limitations. First, the small number of provinces (*N* = 31) and the low frequency of maternal mortality events may reduce the precision of SII and RII estimates. Second, certain health system indicators (e.g., workforce counts) may suffer from measurement error and fail to capture service quality. Third, results reflect associations rather than causal effects, as unobserved confounding cannot be ruled out. Fourth, important socioeconomic variables such as the Gini coefficient were not included and should be incorporated in future analyses. Fourth, our calculation of SII and RII is based on provincial aggregate time-series data, not individual-level records. The indices, therefore, represent the ecological socioeconomic gradient of maternal mortality across different economic periods within a province over time, rather than measuring inequality among individuals. Future research utilizing individual socio-economic and health data is recommended to validate and refine these inequality estimates. Finally, results are specific to China’s provincial context and may not be directly generalizable to other countries. Despite these limitations, the study provides novel insights into the socioeconomic and health system determinants of maternal health inequities in China, highlighting regional heterogeneity and offering evidence-based guidance for equity-oriented policy under the Healthy China 2030 framework.

## 5 Conclusion

Over the past decades, China has made remarkable progress in reducing maternal mortality; however, the issue of maternal health equity has not received sufficient attention. This study examines the relationship between socioeconomic and health system factors and maternal health inequities through the lens of inequality indices. By quantifying provincial-level disparities in maternal health and systematically analyzing their determinants, the study fills an important research gap.

The findings demonstrate that the drivers of reducing average maternal mortality (e.g., healthcare resource inputs) are not necessarily the same as those that reduce health inequities (e.g., wealth level, educational attainment). In fact, simply increasing healthcare resources may not effectively narrow health gaps and may even exacerbate inequities. Instead, economic development, education, and insurance coverage emerged as fundamental in mitigating inequities, especially in central and western provinces, while in eastern provinces, the emphasis should be on improving the efficiency and quality of services.

These results provide empirical evidence to inform the design of more equitable and effective maternal health policies. They highlight the need for regionally differentiated strategies: In central and western provinces, policies should prioritize economic and educational development, insurance expansion, and better workforce distribution through fiscal transfers and infrastructure investment. In eastern provinces, efforts should instead concentrate on efficiency, service quality, and innovation in healthcare delivery.

From a policy standpoint, the findings of this study suggest that reducing maternal health inequities requires a combination of health system reform, workforce rebalancing, and region-specific strategies, rather than reliance on economic growth or hospital expansion alone.

First, strengthening primary health care (PHC) and shifting away from hospital-centric delivery models should be prioritized, particularly in underdeveloped and rapidly urbanizing regions. This includes increasing fiscal support and infrastructure investment in central and western provinces, with special attention to areas experiencing rapid population growth, in order to improve early access, continuity of care, and effective referral for high-risk pregnancies. Second, more balanced allocation of human resources is essential. Policies should promote continuity in midwifery-led and antenatal care services by designing targeted incentive mechanisms, training programs, and career development pathways that encourage healthcare workers to practice in rural and remote settings. Such measures are critical for narrowing gaps in service availability and quality across socioeconomic groups. Third, targeted and differentiated regional interventions are needed. In less-developed regions, expanding insurance coverage, improving benefit packages, and reducing out-of-pocket (OOP) payments are key to alleviating financial barriers and preventing delayed or forgone care. In contrast, in more developed provinces, policy efforts should focus on enhancing service efficiency, quality, and integration within existing health systems.

Together, these strategies underscore that equitable improvements in maternal health depend on integrated, people-centered health system reforms that combine strong PHC networks, financial protection, and region-sensitive policy design.

In sum, the study emphasizes that achieving the fairness objectives of the Healthy China 2030 strategy requires not only lowering average maternal mortality but also addressing inequities across regions and social groups. By highlighting region-specific determinants and providing actionable recommendations, this study offers critical insights for policymakers and contributes to the broader agenda of health equity in China.

## Data Availability

The original contributions presented in the study are included in the article/Supplementary material, further inquiries can be directed to the corresponding authors.
