# Supplementary material for: Socioeconomic and health system determinants of maternal health inequities across Chinese provinces (2009–2021): a multivariate meta-regression analysis of regional heterogeneity in underlying mechanisms
Source: Front Public Health. 2026 Feb 17;14:1722508. doi: 10.3389/fpubh.2026.1722508 (PMC12953505; doi:10.3389/fpubh.2026.1722508)
Supplement: Supplementary file 1 [file Table_1.DOCX]

# Appendix Table S1. Provincial Development Rankings According to CAPGDP in Mainland China

| Rank | Province | CAPGDP | Category |
| --- | --- | --- | --- |
| 1 | Beijing | 115622.87 | High |
| 2 | Shanghai | 113479.05 | High |
| 3 | Tianjin | 98147.08 | High |
| 4 | Jiangsu | 89456.85 | High |
| 5 | Zhejiang | 78884.19 | High |
| 6 | Fujian | 71841.53 | High |
| 7 | Guangdong | 68983.82 | High |
| 8 | Neimenggu | 64944.5 | Upper-middle |
| 9 | Shandong | 61034.09 | Upper-middle |
| 10 | Liaoning | 54888.43 | Upper-middle |
| 11 | Chongqing | 53138.78 | Upper-middle |
| 12 | Hubei | 50007.34 | Upper-middle |
| 13 | Shanxi | 48631.15 | Upper-middle |
| 14 | Jilin | 46119.72 | Middle |
| 15 | Hunan | 43331.0 | Middle |
| 16 | Ningxia | 43306.96 | Middle |
| 17 | Hannan | 41125.53 | Middle |
| 18 | Hebei | 40427.11 | Middle |
| 19 | Xinjiang | 40416.01 | Middle |
| 20 | Henan | 40224.46 | Lower-middle |
| 21 | Anhui | 39407.06 | Lower-middle |
| 22 | Qinghai | 39229.46 | Lower-middle |
| 23 | Sichuan | 38943.27 | Lower-middle |
| 24 | Jiangxi | 38513.94 | Lower-middle |
| 25 | Shanxi | 38055.21 | Lower-middle |
| 26 | Heilongjiang | 37009.51 | Low |
| 27 | Guangxi | 33991.8 | Low |
| 28 | Xizang | 33586.16 | Low |
| 29 | Yunnan | 31549.21 | Low |
| 30 | Guizhou | 30118.7 | Low |
| 31 | Gansu | 26324.44 | Low |
